# Supplementary material for: Characterization and Functional Analysis of a New Calcium/Calmodulin-Dependent Protein Kinase (CaMK1) in the Citrus Pathogenic Fungus Penicillium italicum
Source: J Fungi (Basel). 2022 Jun 25;8(7):667. doi: 10.3390/jof8070667 (PMC9323541; doi:10.3390/jof8070667)
Supplement: Supplementary file 1 [file jof-08-00667-s001.zip › jof-1784980-supplementary.pdf]

Journal of Fungi

MDPI

Supplementary materials

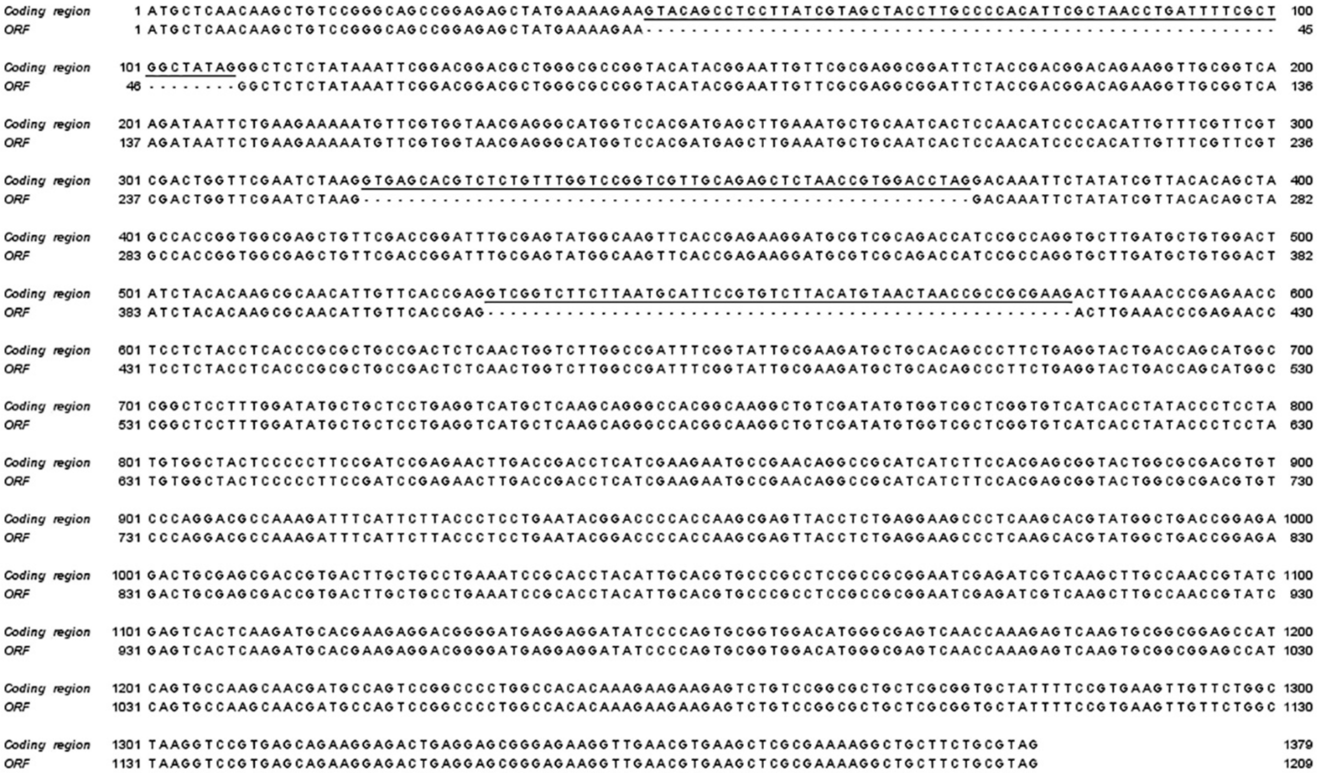

Figure S1. Genomic DNA sequence (coding region) and open reading frame of the PiCaMK1 gene. The underlined sequences indicate introns in the PiCaMK1 gene.

Table S1. Primers applied for gene cloning, knockout, and complementation in the present study.

Primer Name	Sequence (5'→3')	Purpose
PiCaMK1-F	CTCGTATTTCTCTAGTCGC	*PiCaMK1* cloning
PiCaMK1-R	AGTACAAGGCAGGATAGAC	*PiCaMK1* cloning
PiCaMK1-L-F	TAACGTTCCCGTTGTGGCT	Amplification of left arm
PiCaMK1-L-R	CTAGCTAGCGTGACTGACTAGTCACCATTTCAAGCTCATCGTG	Amplification of left arm
[a]*Hyg*-F	GTGACTAGTCAGAAGATGATATTGAAGGAGC	[a]*Hyg* cloning
[a]*Hyg*-R	CTAGCTAGCAAAGAAGGATTACCTCTAAACAAG	[a]*Hyg* cloning
PiCaMK1-R-F	GTGACTAGTCAGTCACGCTAGCTAGGCGAAGACTTGAAACCCGA	Amplification of right arm
PiCaMK1-R-R	GCGATGATGGCTTGAAAATTG	Amplification of right arm
PiCaMK1-[b]Ko-F	CATTGTTTCGTTCGTCGACT	Mutant validation by *PiCaMK1* amplification
PiCaMK1-[b]Ko-R	GAATGCATTAAGACCGACCT	Mutant validation by *PiCaMK1* amplification
PiCaMK1-[c]Diag-F	GTACAGCCTCCTTATCGTAG	Mutant validation by [d]L-H-R amplification
PiCaMK1-[c]Diag-R	CATGCTGGTCAGTACCTCA	Mutant validation by [d]L-H-R amplification
PiCaMK1-COM-F	CCCTCGAGGCAAGAAGATCTACGTGCAG	Complementation plasmid construction
PiCaMK1-COM-R	GGACTAGTGATGTCCATTTGCCATTCCT	Complementation plasmid construction
[e]*Sur*-F	CTAGAGACTAGATCTGTGCC	[e]*Sur* gene cloning
[e]*Sur*-R	AACTTAACCGTGCAGGCCAT	[e]*Sur* gene cloning
PiCaMK1-Probe-F	TGCAATCACTCCAACATCC	Probe yield for southern blot
PiCaMK1-Probe-R	GGCGGTTAGTTACATGTCA	Probe yield for southern blot

Note: [a]*Hyg*-F/R indicates primers to amplify *hygromycin* B (*hyg*)-resistance cassette. [b]*PiCaMK1*-Ko-F/R indicates primers to validate knockout fragments. [c]*PiCaMK1*-Diag-F/R indicates primers to amplify diagnostic fragment. [d]L-H-R indicates the knockout fragment that contains left arm, *hyg*-resistance cassette and right arm. [e]*Sur*-F/R indicates primers to amplify *Sur*-resistance gene that encodes chlorimuron ethyl-degrading enzyme. The underlined sequences indicate restriction sites in the primers (i.e., GCTAGC: *Nhe* I, ACTAGT: *Spe* I, and CTCGAG: *Xho* I).

Table S2. Fungal CaMK genes used in multiple sequence alignments.

Genus and Species	CaMK Gene Accession Number
Penicillium italicum	The present study

Penicillium digitatum	XP_014532050
Penicillium expansum	XP_016598664
Penicillium brasilianum	CEO60329
Penicillium roqueforti	CDM34428
Aspergillus welwitschiae	XP_026623611
Aspergillus flavus	KOC14811
Aspergillus taichungensis	PLN83689
Aspergillus nidulans	XP_660016
Neurospora crassa	AAL14118
Saccharomyces cerevisiae	BAA14383
Saccharomyces cerevisiae	BAA14384

Table S3. Fungal CaMK genes used in phylogenetic tree construction.

Genus and Species	CaMK Gene Accession Number
Penicillium italicum	The present study
Penicillium digitatum	XP_014532050
Penicillium expansum	XP_016598664
Penicillium roqueforti	CDM34428
Penicillium coprophilum	OQE45907
Penicillium arizonense	XP_022485550
Penicillium subrubescens	OKP13003
Aspergillus nidulans FGSC A4	XP_660016
Aspergillus flavus AF70	KOC14811
Aspergillus mulundensis	XP_026603689
Aspergillus welwitschiae	XP_026623611
Aspergillus calidoustus	CEL09411
Aspergillus taichungensis	PLN83689
Aspergillus nomiae NRRL 13137	XP_015409963
Aspergillus japonicus CBS 114.51	XP_025523846
Aspergillus indologenus CBS 114.80	PYI34311
Aspergillus brunneoviolaceus CBS 621.78	XP_025445091
Aspergillus costaricaensis CBS 115574	XP_025544052
Neurospora crassa	AAL14118

Table S4. Primers used in RT-qPCR.

Gene ID	Gene Function Annotation	Primer	Sequence (5'→3')
EKV13547	DNA primase	F	GAAGGTCCCCGAGTTGATCG
		R	AGCCACCACTGCAGACATAC
EKV11776	DNA replication licensing factor Mcm6	F	GATGCTCGCGGTATGTTCCA
		R	TGGCTTCGTGAATAGCGACC
EKV18089	DNA polymerase δ	F	GAGAAGGACCAGCAATGGGA
		R	CCAGCGAATGGCAAGGACAT
EKV08814	DNA polymerase ε	F	TTTCCGTTGACCCTACTGC
		R	CTCCACGATTTGTGCCCTTA
EKV11198	DNA replication licensing factor Mcm3	F	CCGTGGCAAGAATGTGGA
		R	CGAAGAGCAGAATAGGTGGC
EKV17606	Replication factor-a	F	CCCATTCCAAATTGACGG
		R	ACTTCTTGACTTCGCACTCG
EKV16128	DNA replication licensing factor Mcm2	F	GCACTTGTTGCACATTGACCA
		R	TGCCGATGAACGAATCCACA
EKV11033	Nuclear condensing complex	F	ATTCGCAGTGCTACGGGTGT
		R	GCATTTCGGCTAGAGGCT
EKV17483	Cell-cycle checkpoint protein kinase	F	CGAATGGTTTCCGTCAGC
		R	GAGTCACCGAGGCGTCTTT
EKV19093	Nuclear condensin complex Smc2	F	ATGGAACAGGAACACGAG
		R	GCTCTGTCAAGTTGCGTAGG
EKV04214	Replication checkpoint protein	F	GTCGCATTGCAGCGTTTG
		R	TCGGGTAGGAGATGTTAGGTGT
EKV16186	Mitotic spindle checkpoint protein (Mad2)	F	CGCAGTTGAAGGAATGGATG
		R	GGTTAGTTTCGGACTCGGATG
EKV16749	Condensin complex subunit 1	F	GCCAAGTCAAGGTGAAGGG
		R	GAAGAACATGCGAGCCAGAT
EKV04683	Xanthine dehydrogenase (oxidase)	F	CGTCTTCATCTCCGAGACCG
		R	ATATGGCGTAGCCGTTGAGG
EKV19574	Ribonucleoside-diphosphate reductase	F	ATCAGGGTGGTAACAAGCG
		R	GTCCACAAAGCGTAGAAGAGGT
EKV15599	Ribonucleotide reductase RnrA	F	AGCGACCACGAATCAACC
		R	GGAGAATGTGAGACCAGGCA

EKV16890	DNA-directed RNA polymerase III subunit Rpc34	F	TGAAGAACGGCATGACCGAA
		R	GAAGTACTCGCAGGTCTCGG
EKV07940	Adenylate cyclase	F	GACTTCTTTGAACCTCGCC
		R	CAACTTGCCAATCTGAGCC
EKV07761	Uracil phosphoribosyltransferase	F	CATTCCAAGGACACAACGCC
		R	CGGATCGGAGAATTGGCACT
EKV07371	Formamidopyrimidine-DNA glycosylase (H2TH domain)	F	AGGTCGAACTAGTGCCGTTG
		R	CCGTTTGGAATTGGCTGGTG
EKV15299	DNA repair protein RAD1	F	GGGTAAGCTACCCAGCTACA
		R	GAGTGTTGACTGTCCGGAGG
EKV17484	Phosphatidylinositol 4-kinase (STT4)	F	ACGGAGTATTGCTGGTGAG
		R	GCAACGCAACCAAGTAGTGA
EKV05405	Cytochrome c oxidase assembly protein	F	ATCGGATGTCCTCCCTTGGA
		R	CCTGGCGTGACACTGTATGT
EKV18906	F_1/F_0-ATP synthase	F	GGAGGGCCACAATGGAGTTT
		R	GGAGGTTGGGATTAGGTGGC
EKV07272	Aldehyde dehydrogenase	F	CATGGGTGGCACTATGGAT
		R	CAAGATGGGGATGAAGGGA
EKV06483	Cysteine synthase	F	CTGTCAAAACGGCGAGGAA
		R	TCAACAGCCCCACCAACA
EKV18475	Assimilatory sulfite reductase	F	GCCAGAAAGTGACCCCTACC
		R	GACTTGGAGGGCAGCTTCAT
EKV04855	Oligo-1,6-glucosidase (α-amylase or maltase)	F	TGGTTCAAAGAGTCCCGCTC
		R	TTCTTCTGTTGCCTCGTCCC
EKV05685	Glucosamine-6-phosphate deaminase	F	TACTTGTGCAGCGTCATCGT
		R	TCAGTGGGTCGTTTTCTCCG
EKV11299	NADH-cytochrome b_5 reductase	F	GTGGGTAGTCTGGCTCCAAG
		R	ATGTTCTTCTCGGCACCCTC
EKV15950	Chitin synthase	F	AGCTCTTTCGGTGGTTCGAG
		R	CAACCCTACCAGCACTCCAG
EKV13467	DnaJ-related protein	F	TTACGATTCTTGGTCGCGCT
		R	TGACGCGGACTTCGCTATAC
EKV14522	Polyubiquitin binding protein	F	TCAGCTGGACAAGACGCAAT
		R	AGACATTGTGACCGTGACCC
EKV14057	HSP90	F	TTCTACTCCGCCTACCTGG
		R	CCTTGAGGGTGAAAGTGCC
EKV13033	HSP70	F	GAGTTCAAGCGCAAGCACAA
		R	AGAGAGAGTTCGCTTGGCAC
EKV13686	Nuclear protein localization protein	F	AGTCAGGTGCTCAACGACTG
		R	AGTGTCACACCGTCGACTTC
EKV17406	Catabolic 3-dehydroquinase	F	AGCTATGGTGCCGTTCTTG
		R	GGATTGATGATGATCCCGTC
EKV18650	Phospholipase D	F	CACTACCAGGATCGACTCGC
		R	TGATGGCGCAATATGCGTTG
PHI26_NewGene_31	Vacuolar protein sorting-associated protein (VHS domain)	F	ATATCTGTACCGGTGCTGCG
		R	TGGGCTGAACGCTCTTTGAT
PHI26_NewGene_32	Vacuolar protein sorting-associated protein (FYVE-like protein)	F	AGTGTGCCTCAACTCCTTCG
		R	GAAGGCAGTCCTACATCGCA
Internal reference	β-actin	F	TTACCTGATGAAGATCCTCGC
		R	TCTGGATCTCCTGCTCGAAG
